# TIMING AND TREATMENT OPTIONS IN ADULT POLYCYSTIC LIVER DISEASE: A RARE FAMILIAR CASE AS EXAMPLE

**DOI:** 10.1590/0102-672020180001e1411

**Published:** 2018-12-06

**Authors:** Juan Antonio SALCEDA, Ricardo BRACCO, Diego FERNANDEZ

**Affiliations:** 1 Servicio de Cirugía del Hospital Ramón Santamarina, Tandil, Buenos Aires; 2 Sector Cirugía HPB y Trasplante Hepático, Clínica Pueyrredón, Mar del Plata, Buenos Aires, Argentina.

**Keywords:** Polycystic liver disease, Liver Resection, Laparoscopy, Doença policística do fígado, Ressecção hepática, Laparoscopia

## INTRODUCTION

Adult Polycystic Liver Disease (APLD) is a rare affection characterized by multiple cystic lesions of the liver. It may be associated with kidney cysts too and is frequently diagnosed accidentally in images studies as a non symptomatic condition. However, some patients can develop symptoms due to mass effect of multiple and big sized cyst such as abdominal pain, gastric compression, palpable mass and biliary obstruction^1^ ^,^ ^2^

Surgical treatment is the only option available in order to resolve those symptoms and could varies from minimally-invasive surgery to liver transplantation. Once diagnosis is made, surgical strategy and timing to operation can be a challenging situation^3^ ^,^ ^4^ ^,^ ^5^.

## FAMILIAR CASE PRESENTATION

We present a rare familiar case of three sisters with APLD in different grades and also with very different form of presentation in which every case was analyzed separately in order to choose best treatment option and timing to operation as well.

### Case 1

**FIGURE 1:**
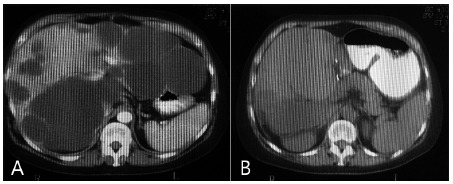
A) CT-Scan shows grade II APLD compromising left later segment and posterior segments of the right lobe; B) post-operative result

A 49 year old woman, very symptomatic, suffering high quadrant abdominal pain, nausea, dyspepsia, dorsal pain and constant episodes of cough leading to impossibility to sleep and rest, conducing to a very decreased quality of life. Cysts were discover by ultrasound and patient was then referred to our service. CT-Scan was later performed (Figure 1). CT showed grade II APLD compromising left lateral segment entirely and big sized cyst located mostly in posterior right segments of the liver. Patient wanted to be operated as soon as possible. Laparoscopic approach was elected and planned surgery was left lateral sectionectomy and fenestration of posterior right cysts. Laparoscopic liver resection and fenestration combined was performed in order to reduce liver mass and relief symptoms.

Laparoscopic left lateral sectionectomy associated with fenestration and partial resection of giants cysts located in right lobe was performed. A very low debit and auto-limited biliary leak was observed and patient was discharged at 8^th^ post-operative day with no further complications.

After more than five years of follow up patient remains without related symptoms and CT-Scan shows only few cysts and hypertrophy of the remanent liver parenchyma. Blood test and liver function were normal.

### Case 2

**FIGURE 2:**
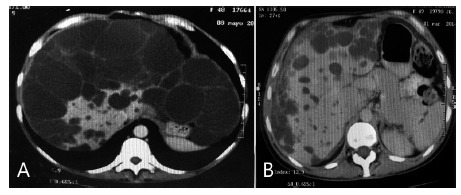
A) Pre-operative CT-Scan shows grade III APLD; B) control CT-Scan shows important hypertrophy of remanent liver parenchyma and several small size cysts remaining

Woman 46 year old with grade III APLD went for consultation as soon as her sister was discharged. She already knew having the same liver condition. CT-Scan and MRI showed more aggressive disease in segments 2 to 8, leaving a portion of caudate lobe with visible parenchyma (Figure 2). Physical exam showed huge palpable mass involving almost all abdominal quadrants. Patient suffered weight loss, nausea and signs of gastric compression. Open liver resection was offered in order to reduce mass but patient refused operation. 

Further regular controls showed increasing of weight loss, abnormal low BMI, and severe bilateral lower limb varicose veins and edema due to IVC compression. Patient still refuse to surgical treatment.

Few months later was admitted in emergency room due to double incarcerated hernia due to intra-abdominal hypertension and both hernias were successfully repaired urgently. Patient then accepted go through liver surgery.

Regarding complete compromise of left lobe and most of the symptoms were related to gastric compression, left open liver resection was planned in association with several non anatomic resection and fenestration in order to make space for the future remanent parenchyma to grow.

Bilateral sub-costal incision was elected and open left lateral sectionectomy associated with several non anatomic resections and fenestration were performed. 

Patient underwent re-laparotomy for lavage due to hemorrhagic liquid in the abdomen causing acute abdominal pain. Then was discharged with no other complications.

After more than three years follow up, she remains without digestive symptoms. She gained weight and has no longer palpable abdominal mass. Inferior members edema disappeared. CT-Scan showed huge hypertrophy of remanent liver and small sized remaining cysts (Figure 2)

### Case 3

Woman 52 year old with severe grade III APLD came in consultation. She was also aware of her familiar condition. Liver transplantation was offered to her in another center but she refused. Previous MRI from another center showed multiple giants cysts compromising the whole abdominal cavity. Cysts reached hypogastric area and both inferior quadrants. Higher segments of the liver seemed to be respected. Globulous deformation of the abdomen was clearly observed. Despite aesthetic and mild abdominal pain symptoms were not as significant as they were in her two sister so she did not want to underwent liver resection too. However she kept coming to consultation every six months.

**FIGURE 3:**
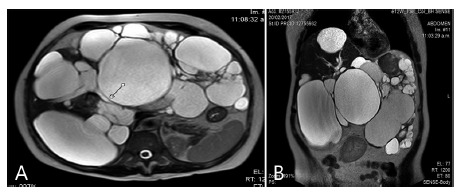
MRI showing severe APLD compressing hepatic pedicle and occupying almost whole abdominal cavity

Two years later she came back presenting jaundice with serum levels of FAL>1700 mg/dl. Bilirrubin 9,8/7,9 mg/dl. New MRI showed cyst compression of the hepatic pedicle including common bile duct and portal vein (Figures 3 A and 3B)

Surgery was soon programed and approach was discussed. We decided laparoscopic approach but being aware room will be very reduced even after pneumoperitoneum. We decided to start by performing aspiration and evacuation of the big lower and anterior cysts to intend reducing the mass and make new space for the laparoscopic procedure. By performing this gesture followed by several fenestration and partial cysts resections space was increased within the abdominal cavity so laparoscopic liver resection could be then performed. Once gallbladder was located, we then identified two cysts that seemed to be responsible for hepatic pedicle compression. Fenestration was done and, then, intra-operative cholangiogram showed no further compression of the common bile duct with adequate passage of contrast to duodenum. Standard cholecystectomy was completed and later laparoscopic left lateral sectionectomy was done in association with several more cyst fenestration. Post-operative course was un-eventful and patient was discharged at 6^th^ day with decreasing levels of FAL and bilirubin. Late follow up imaging control are still to come.

## DISCUSSION

Management of APLD can be a very challenging situation. Radiological findings and also clinical presentation can varies from asymptomatic or very simple cases to severe ones compromising quality of life and developing life threatening events.

This congenital condition rarely compromise liver function. Coagulation and the rest of blood test are usually normal. If not associated with polycystic kidney disease probably will remains stable for long periods. However, clinical presentation may be diverse and its mostly related to mass effect due to hepatomegaly and huge sized cysts. Abdominal pain, palpable mass, gastric compression and weight loss are most frequently observed, but VCI syndrome, intra-abdominal hypertension, bowel obstruction and biliary obstruction can be also observed as in this particular case.

Liver transplantation is the only definitive curative treatment but still remains as an extraordinary option because, as said before, patient usually did not compromise liver function so MELD score used to be low. Listed in low places and lack of donors world wide make liver transplantation as a far and distant option for these patients. Exceptional points can be achieved by demonstration low quality of life but still remains difficult. In addition liver transplantation could be a very aggressive treatment taking into account that liver resection can be performed safely and immediately when needed.

The goal of liver resection associated with multiplies fenestration is to reduce liver mass^6^ ^,^ ^7^. This end point of this tactic will be achieving not only relief of the symptoms but also will provide new space into the abdominal cavity that will allow liver parenchyma to grow and increase volume.

Planning the strategy for a successful liver resection in APLD is not simple. Current anatomy pedicles and vessels have often changed their places because of cysts. Recognizing structures in this altered situation can be challenging in imaging studies and barely impossible during surgery. 

Even normal parenchyma is replaced by cysts, vessels and bile ducts are still somewhere within cysts walls, that can make liver transection really difficult and a prepense situation for bleeding or bile leaking. 

Planning type of hepatic resection is not the only thing to discuss once surgical treatment is decided. Timing to surgery is often an issue that can remain unclear. Most of these patients must go to a programed procedure. As every chronic disease patients are used to be tolerant with symptoms and some times they may be not decided enough for an aggressive treatment. Usually acceptance came along when symptoms became stronger or less tolerated. Long delay between diagnose and surgical treatment was already published in different series. 

Concerning about the approach, laparoscopy seems to be safe and feasible in selected patients. It provides all advantages of minimally invasive access without compromising the standard technique. In experienced hands also grade III can be benefited with this approach^8^ ^,^ ^9^ ^,^ ^10^. 

In summary, although is not a curative treatment, liver resection associated or not with fenestration could be the best option for patient with APLD. In selected patient this strategy would achieve palliation of all symptoms and also provide space allowing hypertrophy of remanent liver parenchyma.
